# Later‐life cognitive function at the intersection of gender and occupation across Harmonized Cognitive Assessment Protocols (HCAPs) in the United States, Chile, Mexico, India, and South Africa

**DOI:** 10.1002/alz.70923

**Published:** 2025-11-28

**Authors:** Sneha Sarah Mani, Katrina L. Kezios, Magdalena Delaporte, Tsai‐Chin Cho, L. Paloma Rojas‐Saunero, Jennifer Weuve, Justina Avila‐Rieger, Lindsay C Kobayashi

**Affiliations:** ^1^ Department of Epidemiology Johns Hopkins School of Public Health Baltimore Maryland USA; ^2^ Department of Epidemiology School of Public Health, Boston University Boston Massachusetts USA; ^3^ Population Studies Center University of Pennsylvania Philadelphia Pennsylvania USA; ^4^ Department of Epidemiology School of Public Health, University of Michigan Ann Arbor Michigan USA; ^5^ Department of Epidemiology UCLA Fielding School of Public Health Los Angeles California USA; ^6^ Department of Epidemiology Mailman School of Public Health, Columbia University New York New York USA

**Keywords:** cross‐national comparisons, dementia, gender, later‐life cognition, MAIHDA, occupation, older adults, social norms

## Abstract

**INTRODUCTION:**

We examined how gender and lifetime occupational skill intersect cross‐nationally to influence later‐life cognitive function.

**METHODS:**

Data were from 11,450 participants ≥50 years of age from Harmonized Cognitive Assessment Protocols (HCAPs) in the United States, Chile, Mexico, India, and South Africa. We used multilevel analysis of individual heterogeneity and discriminatory accuracy (MAIHDA) to decompose the variance in cross‐nationally harmonized later‐life cognitive function.

**RESULTS:**

In adjusted linear mixed‐effects models, variance between strata accounted for 15.7% of the overall variance in cognitive function, attributable to the intersectional effects of gender and lifetime occupational skill across the five countries. After adding fixed effects of the individual factors of gender, lifetime occupational skill, and country to the model, their intersectional effects accounted for 4.4% of overall variance.

**DISCUSSION:**

Our findings suggest that life course experiences at the intersection of gender and lifetime occupation across countries are associated with later‐life cognitive function, beyond their individual effects.

**Highlights:**

- We investigated how the intersection of gender and lifetime occupational skill influences later‐life cognitive function across countries.
- Applying the multilevel analysis of individual heterogeneity and discriminatory accuracy (MAIHDA) framework, we show that the intersectional effects of these factors accounted for 15.7% of the overall variance in later‐life cognitive function, with residual intersectional effects of 4.4% even after adjusting for their independent contributions.
- These findings highlight that life course experiences shaped by gender, occupation, and country of residence are crucial for understanding differences in later‐life cognitive function.

## 1 BACKGROUND

In 2023, a total of 55 million people worldwide were living with dementia, with low‐ and middle‐income countries (LMICs) comprising over 60% of these cases.^1^ Globally, dementia prevalence is higher in women than men, especially in LMICs,^2^ although there is country‐level variation.^2^ A recent meta‐analysis of data on nearly one million older adults across 43 countries showed that gender differences in life expectancy and education largely contribute to the higher dementia prevalence in women compared to men.^3^ However, other social risk factors for dementia that are shaped by gendered social norms and structures in different countries may additionally help explain cross‐national heterogeneity in gender differences in dementia prevalence.^2^, ^3^

Occupation is a key life course pathway through which individuals may learn new skills and engage in complex mental activities and human interactions that benefit later‐life cognition; however, not all work is cognitively protective.^4^, ^5^, ^6^, ^7^, ^8^, ^9^ Workplace environments are heterogeneous, including cognitively beneficial (e.g., cognitive stimulation, physical activity, social support, access to health care) and/or cognitively harmful components (e.g., neurotoxicant chemical exposure, heat, noise, psychological stress).^10^ These environments are also unequally patterned by gender.^11^, ^12^ In general, compared to men, women face more occupational stressors like unequal pay, workplace harassment, and lower job control, and have fewer opportunities for entry, advancement, and promotion.^13^ However, women are also less likely than men to work jobs characterized by cognitively harmful physical workplace hazards such as noise or chemical exposures.^14^ These differences complicate our understanding of the effects of occupation on cognition for men and women.

The relationship between occupation and cognition for men and women also likely varies across different country contexts, although few studies have examined how gender and occupation jointly influence cognition across countries, especially not across high‐, middle‐, and low‐income countries. Yet, globally, women's ability to enter/remain in the labor force and their access to cognitively beneficial workplace opportunities depends on country‐level economic policies (e.g., equal pay, paid family leave), gendered and patriarchal social norms (e.g., attitudes about women receiving education, mobility restrictions, expectations that women perform domestic responsibilities), and gendered workplace power structures (e.g., hiring and promotion practices that preferentially benefit men, misogyny).^12^, ^15^, ^16^ Several global gender equality indices quantify aspects of these dynamics, and these indices help provide context for geographical variations in labor force participation and occupation by gender and their impact on cross‐national differences in cognition.^17^ For example, the 2023 Gender Social Norms Index reports that between 2010 and 2014, 75% of men and women in India held biased attitudes about women's roles as business executives and believed that men have more of a right to work than women, compared with 29% in Mexico and 15% in the United States. ^17^ Correspondingly, more women in India have never participated in the labor force over their life course, a risk factor for cognition and dementia, compared with women in Mexico or the United States.^4^

The complex ways that gender and occupational experiences interact to influence later‐life cognition may vary across country contexts, implying that gender, occupation, and country should be considered jointly for a comprehensive understanding of their effects on cognition. Intersectionality is a relevant theoretical framework to guide such research. Intersectionality contends that because people's lived experiences are differently shaped across their life courses by interwoven systems of oppression and power structures that tacitly or implicitly advantage specific groups and disadvantage others, the aspects of social identity shaped by these systems, such as gender and class, should not be studied in isolation.^18^, ^19^ Therefore, in this study, we used harmonized, population‐based data on men and women from the United States, Chile, Mexico, India, and South Africa, a and employed a statistical method proposed for intersectionality research, multilevel analysis of individual heterogeneity and discriminatory accuracy (MAIHDA), to examine how later‐life cognition is patterned at the intersection of gender and lifetime occupational skill level across countries. The use of MAIHDA in this cross‐national context can offer insights into how later‐life cognition is patterned across diverse social, cultural, and policy contexts. This information may help identify potential mechanisms and inform policies that are tailored to the complex social realities of each country.

**RESEARCH IN CONTEXT**

1. **Systematic review**: Gender differences in dementia prevalence vary cross‐nationally. We searched PubMed and Google Scholar to identify the literature on social determinants of cross‐national gender differences in dementia. Globally, life expectancy and education largely contribute to higher dementia prevalence in women compared to men, but other social risk factors shaped by gendered social norms in different countries, like occupation, may explain remaining differences.
2. **Interpretation**: In cross‐nationally harmonized data, we observed intersectional inequities in later‐life cognitive function by gender and lifetime occupation across the United States, Mexico, Chile, South Africa, and India. This finding may signal important societal processes operating at the country level that differentially influence how gender and occupation shape later‐life cognition.
3. **Future directions**: Future studies should investigate the roles of structural gender equality, country‐level gender norms, and different types of gendered work experiences on later‐life cognition and replicate our findings in other low, middle, and high‐income countries.

## 2 METHODS

### 2.1 Study population

We used data from the Harmonized Cognitive Assessment Protocol (HCAP) sub‐studies of the U.S. Health and Retirement Study (HRS) and its harmonized International Partner Studies in South Africa, India, Mexico, and Chile. The source data and their survey years are: U.S. HRS‐HCAP (2016; ages ≥65 years),^20^ Health and Ageing in Africa: A Longitudinal Study of an INDEPTH Community in South Africa (HAALSI) (HAALSI‐HCAP; 2016; ages ≥50 years),^21^ Harmonized Diagnostic Assessment of Dementia for the Longitudinal Aging Study in India ‐  (LASI‐DAD; 2017‐2019; ages ≥60 years),^22^ Mexican Cognitive Aging Ancillary Study (Mex‐Cog; 2016; ages ≥55 years),^23^ and Chile Cognitive Aging Study (CHILE‐COG; 2019; ages ≥60 years).^24^ We excluded 847 participants (7%) who had missing data on cognitive factor scores, lifetime occupation, or completed schooling level. The study sample included 6619 women and 4831 men (11,450 participants total), comprising 3077 participants from the United States, 582 from South Africa, 3747 from India, 2014 from Mexico, and 2030 from Chile (Figure SA1).

### 2.2 Gender, occupation, and country strata

The exposure variable was the intersectional stratum of gender, main lifetime occupational skill level, and country of residence (hereafter, “country”). In each study, participants self‐reported their sex/gender as male or female. Consistent with prior HCAP work, we refer to this variable as “gender.”^25^ Main lifetime occupation skill level (hereafter, “occupation”) was based on self‐reported occupation and harmonized across countries according to the 2008 International Standard Classification of Occupations (ISCO‐08) as: never worked, Level 1 (jobs involving routine physical/manual labor), Level 2 (jobs involving information storage or ordering, operating, maintaining, and repairing equipment), and Level 3 or 4 (referred to as Level 3+; jobs involving complex technical skills or problem‐solving, creativity, and decision‐making).^26^ More details are in the Supplementary Appendix, and Kobayashi et al.^4^ provide detailed descriptions of the crosswalks between occupation titles and ISCO‐08 categories. Table A1 shows the distribution of occupation by country and gender. Finally, to examine cross‐national differences in the patterning of later‐life cognition at the intersection of gender and lifetime occupation, we created 40 strata representing all combinations of the three variables (i.e., five countries, two gender categories, and four occupational skill categories).

### 2.3 Cognitive function

The outcome was a cross‐nationally harmonized general cognitive function (GCF) factor score that was developed previously using confirmatory factor analysis on the HCAP neuropsychological battery of memory, executive function, orientation, and language. ^25^ The cognitive test items are comparable in all countries, with linguistic, educational, cultural, and other adaptations as needed.^27^ The GCF score was standardized to the HRS‐HCAP distribution and had a mean = 0 and standard deviation (SD) = 1. Hence, a GCF score of one refers to one SD unit higher than the average cognitive function of the HRS‐HCAP sample population.

### 2.4 Covariates

Covariates of interest included age (years; linear and quadratic terms), participant's highest level of educational attainment, and whether participant's mother and father attended school (yes; no; and missing).^4^ Participant educational attainment was harmonized according to the 2011 International Standard Classification of Education (ISCED)^28^ to compare education qualifications cross‐nationally. Categories included no formal education, primary education, lower secondary education, upper secondary education, and any college (we added “no formal education” for participants who did not attend school). We adjusted for participant and parental education to disentangle the contributions of gender, occupation, and country to variance in later‐life cognition from that of education (a known contributor to cross‐national gender differences in dementia prevalence). Finally, we did not include minority racial/ethnic status and marital status as covariates in this analysis. Because of the context‐specific nature of racialization and other related forms of marginalization across countries and resulting differences in the variables representing race and ethnicity across countries (e.g., race in the United States, caste in India), we encountered difficulties in meaningfully harmonizing a variable for minority racial/ethnic status across countries. Therefore, in the main analyses, we neither include minority racial/ethnic status as an intersecting factor nor adjust for it as a covariate. However, in supplemental analyses, we adjusted for the best available cross‐nationally harmonized measure of minority racial/ethnic status to evaluate its impact on our findings. We also did not include marital status because the proportion of respondents who were ever married was very high across all countries and occupational skill levels, generally exceeding 90% (Table 1).

**TABLE 1:** Summary statistics by skill level occupation (*N* = 11,450).

	USA		Mexico		India		South Africa		Chile	
	Men	Women	Men	Women	Men	Women	Men	Women	Men	Women
**Skill Level 1**										
Cognitive score, mean (SD)	−0.1 (0.9)	−0.6 (1.0)	−1.1 (0.9)	−1.2 (0.9)	−1.3 (0.8)	−1.8 (0.7)	−1.2 (0.7)	−1.3 (0.7)	−0.8 (0.9)	−0.7 (0.9)
Age, mean (SD)	73.8 (6.7)	75.6 (8.0)	69.9 (9.3)	68.2 (9.1)	69.2 (6.8)	68.4 (7.2)	70.0 (10.9)	67.3 (11.2)	69.9 (8.6)	69.9 (7.9)
Schooling attainment, %										
None	4.8	7.3	64.1	68.8	60.0	89.9	89.1	80.8	42.2	41.5
Primary	6.0	20.8	22.3	22.1	14.7	7.0	5.9	10.2	33.3	31.3
Lower secondary	10.7	21.2	11.2	8.1	10.9	1.3	4.1	4.9	13.4	13.5
Upper secondary	66.1	46.2	1.6	1.0	13.2	1.5	0.9	2.6	10.3	12.5
Any college	12.3	4.5	0.8	0.0	1.2	0.3	0.0	1.4	0.7	1.1
Parental education, %										
No	12.1	18.4	73.7	79.2	87.6	86.0	90.6	83.0	51.7	52.3
Yes	80.7	69.2	13.7	11.0	10.8	11.1	9.4	16.3	27.2	30.9
Missing	7.2	12.4	12.5	9.8	1.6	2.9	0.0	0.7	21.1	16.8
Marital status, %										
Never married	2.3	8.0	3.0	6.7	1.9	0.4	9.4	3.7	13.0	15.5
Ever married	97.7	92.0	97.0	93.3	98.1	99.6	90.6	96.3	84.4	81.2
Missing	0.0	0.0	0.0	0.0	0.0	0.0	0.0	0.0	2.6	3.3
**Skill Level 2**										
Cognitive score, mean (SD)	−0.2 (0.9)	0.0 (1.0)	−0.6 (0.9)	−0.6 (1.0)	−1.2 (0.8)	−1.8 (0.7)	−1.0 (0.8)	−1.4 (0.8)	−0.5 (0.9)	−0.3 (0.8)
Age, mean (SD)	75.7 (7.0)	75.8 (7.0)	67.5 (8.6)	67.0 (8.6)	69.9 (7.5)	69.5 (7.3)	69.8 (10.0)	68.8 (12.2)	70.7 (7.8)	69.5 (7.9)
Schooling attainment, %										
None	3.2	3.0	42.0	38.8	53.2	87.9	67.2	85.3	27.5	20.2
Primary	7.9	4.8	27.3	23.7	16.8	5.8	17.6	4.9	32.4	28.3
Lower secondary	9.9	12.3	15.9	30.3	9.8	1.2	7.7	0.0	16.5	23.2
Upper secondary	61.2	67.8	6.8	3.4	15.5	3.8	7.5	9.8	19.0	22.6
Any college	17.9	12.2	8.1	3.8	4.6	1.3	0.0	0.0	4.6	5.6
Parental education, %										
No	10.6	10.2	72.4	64.0	82.6	88.1	86.6	74.8	44.6	40.1
Yes	82.5	84.0	17.6	29.2	15.3	10.0	13.4	25.2	34.7	44.8
Missing	6.9	5.7	10.0	6.8	2.1	1.9	0.0	0.0	20.6	15.1
Marital status, %										
Never married	3.0	3.7	3.3	7.6	0.9	0.1	3.3	13.7	7.5	18.2
Ever married	97.0	96.2	96.7	92.4	99.1	99.9	96.7	86.3	90.1	79.6
Missing	0.0	0.2	0.0	0.0	0.0	0.0	0.0	0.0	2.4	2.2
**Skill Level 3+**										
Cognitive score, mean (SD)	0.4 (0.8)	0.4 (1.0)	0.1 (0.8)	0.2 (0.8)	−0.5 (0.7)	−0.3 (0.9)	−0.5 (0.8)	−0.6 (0.9)	0.2 (0.8)	0.2 (0.9)
Age, mean (SD)	76.5 (7.3)	75.7 (7.0)	67.4 (8.4)	67.2 (9.2)	70.3 (7.1)	68.9 (8.8)	64.5 (8.2)	59.1 (11.8)	69.6 (7.7)	70.3 (8.3)
Schooling attainment, %										
None	0.2	0.6	8.1	2.7	8.0	13.7	43.6	34.6	5.5	5.0
Primary	0.8	0.3	9.7	7.7	6.4	5.5	22.2	21.0	8.4	10.1
Lower secondary	1.2	2.4	17.8	27.9	9.3	4.4	6.9	11.1	11.0	10.8
Upper secondary	33.8	39.5	7.4	5.9	39.0	35.4	0.0	11.1	23.2	22.7
Any college	64.1	57.2	57.0	55.7	37.4	41.0	27.4	22.2	51.8	51.4
Parental education, %										
No	2.2	4.0	49.7	43.7	58.0	44.9	93.1	77.8	23.3	19.0
Yes	94.5	93.6	43.7	51.7	38.1	50.8	6.9	22.2	66.8	78.4
Missing	3.3	2.4	6.6	4.6	3.9	4.4	0.0	0.0	10.0	2.6
Marital status, %										
Never married	3.0	3.3	5.9	10.6	2.3	11.4	6.9	22.2	5.3	21.1
Ever married	97.0	96.5	94.1	89.4	97.7	88.6	93.1	77.8	93.9	75.5
Missing	0.0	0.2	0.0	0.0	0.0	0.0	0.0	0.0	0.8	3.4
**Never worked**										
Cognitive score, mean (SD)	−0.6 (0.9)	−0.7 (1.0)	−1.3 (0.9)	−1.1 (0.9)	−1.4 (0.9)	−1.6 (0.8)	−1.4 (0.7)	−1.4 (0.8)	−1.7 (0.7)	−0.9 (0.9)
Age, mean (SD)	73.4 (6.3)	77.9 (9.2)	67.9 (8.9)	67.2 (9.2)	70.5 (8.0)	69.8 (8.3)	69.1 (10.7)	69.5 (11.7)	79.3 (10.8)	74.1 (8.8)
Schooling attainment, %										
None	6.1	19.4	58.5	64.6	63.4	74.3	84.5	85.4	41.7	41.5
Primary	13.8	21.3	31.3	23.0	11.3	11.2	7.3	6.6	37.3	34.1
Lower secondary	15.6	21.1	5.1	10.8	10.0	5.6	0.0	4.8	4.8	11.8
Upper secondary	52.6	29.5	5.2	0.5	11.2	8.0	0.0	1.5	5.2	10.5
Any college	12.0	8.6	0.0	1.1	4.1	0.9	8.1	1.5	11.0	2.1
Parental education, %										
No	8.1	23.0	74.0	75.7	85.5	78.0	82.0	85.5	36.9	55.2
Yes	62.0	60.4	10.5	15.2	11.1	18.9	18.0	13.9	47.5	27.4
Missing	29.9	16.6	15.5	9.1	3.4	3.2	0.0	0.5	15.5	17.4
Marital status, %										
Never married	13.9	6.5	5.1	2.5	1.8	0.5	23.5	2.4	29.2	8.1
Ever married	86.1	93.5	94.9	97.5	98.2	99.5	76.5	97.6	70.8	90.3
Missing	0.0	0.0	0.0	0.0	0.0	0.0	0.0	0.0	0.0	1.5

*Note*: Data Sources for each country: South Africa—Health and Ageing in Africa: A Longitudinal Study of an INDEPTH community in South Africa; USA—Health and Retirement Study ‐ Harmonized Cognitive Assessment Protocol; India—Harmonized Diagnostic Assessment of Dementia for the Longitudinal Aging Study in India; Mexico—Mexican Cognitive Aging Ancillary Study; Chile—Chile Cognitive Aging Study. Estimates include sampling weights.

### 2.5 Statistical analyses

We examined participant characteristics within each gender, occupation, and country stratum. We then used the MAIHDA approach to investigate cognitive function at the intersection of gender and occupation across countries. In contrast to fixed‐effects models,^29^ which focus on estimating mean differences in outcomes between groups, MAIHDA is a multilevel modeling framework that emphasizes the variation in an outcome additionally explained by the intersecting effects of factors that constitute the intersectional strata, above and beyond the variation explained by the individual effect of each factor in isolation.^30^, ^31^, ^32^, ^33^, ^34^, ^35^

Following the MAIHDA protocol, we fitted two linear mixed‐effects models with GCF score as the outcome. First, we fitted a “null” model with the 11,450 individual CGF observations at Level 1 clustered within the 40 intersectional strata defined by gender, occupational skill, and country of residence at Level 2. This model adjusted for the fixed effects of the covariates listed above and specified a random intercept for intersectional strata. This null model decomposes the proportion of the total variance in GCF scores that is attributable to between‐stratum variance and between‐person variance. It computes the variance partition coefficient (VPC) as the between‐stratum variance divided by the total variance in CGF scores. The VPC is a summary measure that tells us how much of the total variance in GCF scores is attributable to differences at the strata level rather than the individual level (e.g., if the VPC was 0%, then knowing what stratum a person belongs to would tell us nothing about their predicted GCF score).^33^ We then fitted a “main effects” model by additionally adjusting for each variable that formed the intersectional strata, gender, occupation, and country of residence, in the fixed effects portion of the model. We similarly computed a VPC for this main effects model, which indicates the proportion of total variance in GCF scores that remained attributable to differences between strata after accounting for their individual effects on GCF.^33^ We also calculated the proportional change in variance (PCV) between the null model and main effects model, to quantify the degree of between‐stratum variance that was explained by adjustment for country, gender, and occupation. The remaining *unexplained* between‐stratum variance (1‐PCV) in the main effects model was attributed to intersectionality between the three variables.

All analyses incorporated HCAP sampling weights (accounting for sampling probabilities in each country's parent cohort study and their HCAP subsamples) to obtain estimates representative of the target population within each country. We scaled the sampling weights for each country to be proportional to their HCAP sample size.^4^ Moreover, we estimated the predicted GCF score and the random effects for each stratum for the main effects model and used bootstrap methods to construct the 95% confidence interval for the estimates. All analyses were conducted in STATA 17 and R version 4.3.3.

## 3 RESULTS

Sample sizes for the strata ranged from 10 (South African, women, Level 3+ occupation stratum) to 1215 (Indian, women, never worked stratum) (Table 2). Table 1 and Table A1 describe the characteristics of the sample by gender, occupation, and country. Briefly, the average age for HCAP participants in Mexico, India, South Africa, and Chile was similar (≈70 years), whereas U.S. participants were older (≈75 years), and no consistent age patterns were observed across countries by gender and occupation. Across countries, participant and parental educational attainment were higher in higher skilled occupation strata; in some countries, women were less likely to have attained any college education than men across all occupational skill levels 1–3+ (United States, Mexico); in others, this education disparity was observed only at Level 3+ occupations (South Africa, Chile).

**TABLE 2:** Sample size by stratum.

	USA		Mexico		India		South Africa		Chile	
	Men	Women	Men	Women	Men	Women	Men	Women	Men	Women
All	1,230	1,847	818	1,196	1,689	2,058	222	360	872	1,158
Skill occupation level										
Level 3+	446	608	114	110	186	42	15	10	113	126
Level 2	629	1,072	333	336	1,073	544	59	25	604	393
Level 1	120	65	352	396	333	257	134	171	138	349
Never worked	35	102	19	354	97	1,215	14	154	17	290

*Note*: Data Sources for each country: South Africa—Health and Ageing in Africa: A Longitudinal Study of an INDEPTH Community in South Africa; USA—Health and Retirement Study ‐Harmonized Cognitive Assessment Protocol; India ‐ Harmonized Diagnostic Assessment of Dementia for the Longitudinal Aging Study in India—; Mexico—Mexican Cognitive Aging Ancillary Study; Chile—Chile Cognitive Aging Study. Estimates include sampling weights.

Intersectional strata defined by gender and occupation across countries contributed to observed variance in later‐life cognitive function (Table 3). In the null model, the between‐stratum variance was 0.085 and the within‐stratum variance was 0.459, meaning 15.7% (VPC) of the total variance in GCF scores existed between the intersectional strata. In the main effects model, the between‐stratum variance was 0.021, and the VPC was 4.41%. The PCV was 75%; thus, 25% of the between‐stratum variance was left unexplained by adjustment for the fixed effects of gender, occupation, and country, and is attributed to their intersectional effects.

**TABLE 3:** MAIHDA model results for harmonized cognitive factor scores.

	Null	Main
Country (ref: USA)		
Mexico		−0.216^***^ (0.065)
India		−0.616^***^ (0.078)
South Africa		−0.304^***^ (0.083)
Chile		−0.112 (0.082)
Female		0.004 (0.047)
Skill level of lifetime occupation (ref: never worked)		
Level 1		0.137 (0.075)
Level 2		0.257^***^ (0.076)
Level 3+		0.384^***^ (0.079)
Schooling attainment (ref: no formal schooling)		
Primary	0.568^***^ (0.034)	0.568^***^ (0.034)
Lower secondary	0.793^***^ (0.047)	0.793^***^ (0.047)
Upper secondary	1.082^***^ (0.062)	1.082^***^ (0.062)
Any college	1.393^***^ (0.065)	1.393^***^ (0.065)
Parental education (ref: no formal schooling)		
Some formal education	0.169^***^ (0.022)	0.169^***^ (0.022)
Missing information	−0.060 (0.033)	−0.060 (0.033)
Age	0.053^**^ (0.019)	0.053^**^ (0.019)
Age^2^	−0.001^***^ (0.000)	−0.001^***^ (0.000)
Constant	−1.992^***^ (0.596)	−1.939^**^ (0.609)
Between‐group variance	0.085	0.021
Within‐group variance	0.459	0.459
Variance partition coefficient (%)	15.700	4.409
Proportional change of the variance (%)		75
*N*	11.450	11.450

*Note*: Sources: Health and Retirement Study‐ Harmonized Cognitive Assessment Protocol, Mexican Cognitive Aging Ancillary Study, Harmonized Diagnostic Assessment of Dementia for the Longitudinal Aging Study in India, Health and Aging in Africa: Longitudinal Study of an INDEPTH Community in South Africa, Chile Cognitive Aging Study. Harmonized score of global cognitive function was standardized to the distribution of the unweighted HRS‐HCAP sample, which had a mean of 0 and a standard deviation of 1.

** *p < *0.01.

*** *p < *0.001.

**FIGURE 1 alz70923-fig-0001:**
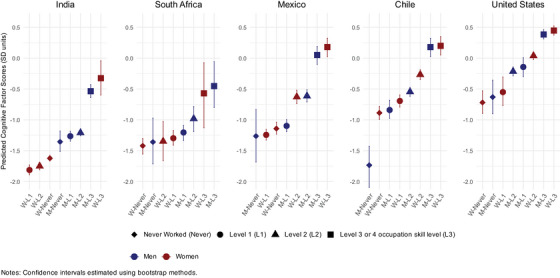
Predicted values of cognitive factor score by stratum.

The main‐effects model provides additional information about the fixed effects of country, gender, and occupation on later‐life cognitive function (Table 3, column 2). On average, compared with the United States, GCF scores were lower in Chile, Mexico, South Africa, and India (in order of increasing magnitude of association); were similar for men and women; and were higher for those with higher occupational skill levels (Table 3). Figure 1 plots the mean predicted GCF scores for each intersectional stratum from the main effects model with 95% confidence intervals (see also Table A2), grouped by country; the predictions incorporate information from both the fixed and random components of the model. Across all strata, the highest mean GCF scores were observed for men and women working in Level 3+ occupations in the United States, Chile, and Mexico (all with mean GCF scores >0). In all countries except South Africa, the women with Level 3+ occupations had slightly higher mean GCF scores than men in the Level 3+ strata, although this gender difference was minimal in all countries. Lower mean GCF scores were observed for the strata with men and women who never worked, and, in general, a higher occupational skill level was associated with higher GCF scores. Exceptions to this trend were observed among both Indian and South African women who never worked and had Level 1 and Level 2 occupations, where gains in mean GCF scores with higher occupation levels were minimal.

**FIGURE 2 alz70923-fig-0002:**
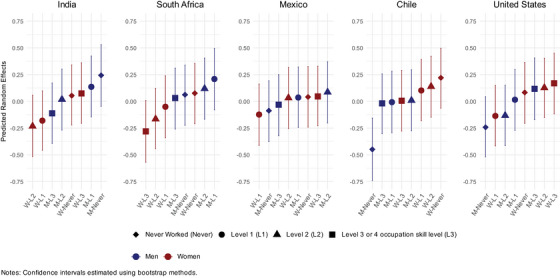
Random effects estimates by stratum.

Figure 2 plots the stratum‐level residual from the main effects model grouped by country, highlighting which intersectional strata had predicted GCF scores that deviated from expected patterns based on fixed effects (with the magnitude of deviations shown in rank order from most negative to most positive). For example, mean GCF scores for the stratum of men in Chile who never worked were significantly lower than expected. In comparison, Chilean women who never worked had better than expected cognitive function. Additional analyses, including minority racial/ethnic status as a covariate, showed similar results. In the null model, the between‐stratum variance was 0.086 and the within‐stratum variance was 0.45. In the main effects model, the between‐stratum variance was 0.022, and the VPC was 4.58% (Table A3).[Fig alz70923-fig-0001], [Fig alz70923-fig-0002]

## 4 DISCUSSION

This study examined how gender and occupation may intersect to shape men's and women's later‐life cognitive function across countries. Overall, in cross‐nationally harmonized data,^25^ cognitive scores were higher on average in the United States than in most other countries, were similar for men and men, and were higher as occupational skill level increased. Using the MAIHDA approach, we observed that 25% of the variance in cross‐national cognitive scores between intersecting strata of gender and occupation was attributable to intersectional effects, not the independent effects of each stratum factor. We observed large‐scale inequities across strata in mean cognitive scores, and the intersectional stratum‐level residuals identified strata for which cognitive scores especially deviated from expected values based on fixed effects. These findings may have been otherwise concealed in a conventional analysis focusing on the independent effects of gender, occupation, and country, highlighting key insights offered by the MAIHDA approach, it moves beyond the focus on group averages and focuses on heterogeneity across strata.^36^

Our results align with existing research on lifetime occupations and later‐life cognitive function, which consistently shows positive gradients between occupational skill/complexity and later‐life cognitive function,^3^, ^4^, ^34^, ^37^, ^38^, ^39^, ^40^, ^41^, ^42^, ^43^ as well as some evidence of gender differences in these relationships. Although the exact mechanisms underlying the influence of occupation history on cognitive outcomes are still being explored, existing evidence suggests that occupational environments may influence cognitive health in later life in both protective and deleterious directions, through cognitively stimulating activities and social interactions, as well as physically or psychosocially straining exposures such as heavy manual labor, noise or chemical exposures, or high stress. ^37^, ^38^, ^39^, ^44^, ^45^, ^46^, ^47^, ^48^ Cognitively complex work may also promote the accumulation of cognitive reserve by engaging neural networks across the lifespan, buffering the effects of neurodegeneration.^49^, ^50^ Taken together, this evidence suggests that whether occupation contributes to maintaining or impairing later‐life cognition depends not only on the duration of labor participation but also on the cognitive stimulation, psychosocial demands, and physical exposures embedded in one's lifelong work history.

Our findings also align with recent research showing that levels of gender inequality within and across countries may contribute to cognitive outcomes. For example, Jain et al. examined cross‐state variation in cognitive performance for men and women in India, observing that although women, on average, score lower on cognitive assessments than men, the magnitude of this gender gap is greatest in states with high gender inequality.^51^ Across 43 countries, Huque et al. similarly observed higher dementia rates for both men and women in countries with higher levels of country‐level indicators of gender inequality.^3^ Across 27 countries, Bonsang et al. also found that in countries with more equal gender‐role attitudes, the cognitive performance of older women and men was higher, but women's cognition especially benefited from living in more gender‐egalitarian contexts;^52^ it is important to note that this finding was not entirely explained by women's educational attainment or labor‐force participation, consistent with our observation of an intersectional effect of gender and occupation across countries.

We also documented divergent patterns in the residual GCF scores for some strata. We observed lower GCF scores than expected for men in Chile and the United States who never worked and women in higher‐skilled occupations in South Africa, although the deviation was only statistically significant for the stratum of Chilean men who never worked. Still, from an intersectional perspective, identifying unique properties among strata with divergent findings may help inform understanding of the variation in cognitive health attributable to different gendered life‐course work experiences across countries. For example, Chile and the United States are high‐income countries that score better than the global average on gender equality indices based on women's basic legal rights and economic opportunities,^17^, ^53^ but are still characterized by deeply ingrained patriarchal attitudes and expectations about men's and women's place in the workforce (e.g., men as “breadwinners”).^54^ In similar high‐income settings, prior research suggests that “norm‐deviating” employment (e.g., men not working, men working part‐time) may be associated with worse cognitive function.^11^ In addition, in India, a U‐shaped relationship between education and women's labor force participation has been observed, women with very low and very high schooling levels and living in rural areas are more likely to work, whereas women with some secondary schooling have the lowest labor force participation.^15^ In economic terms, this is similar to an income effect where women opt out of the labor force when incomes increase and enter again when the opportunity cost of not working is very high.^13^ This selection into the labor force might explain why we see lower cognitive function among women in India who worked in occupations at skill Levels 1 or 2, compared to the stratum of women in India who never worked. Furthermore, these women are most likely to be in occupations with a higher manual workload and experience greater physical strains that negatively impact cognitive and brain health.^55^, ^56^ This flags that not all occupational experiences may be beneficial for later‐life cognitive health.

A key limitation of this study is that lifetime occupational skill level does not capture differences in employment intensity over the life course, such as working part‐time versus full‐time, which may be associated with differential cognitive benefits, particularly for women.^11^, ^41^, ^57^ However, lifetime occupation skill level could be reasonably harmonized using an international coding schematic (ISCO‐08). In addition, we did not examine the type of work undertaken by participants, which may differ for men and women or across countries within given occupational skill levels.

Understanding how the cumulative intensity, type, and complexity of work, as well as exposure to adverse environmental, physical, and psychological occupational hazards, influence the patterning of cognition we observed is an important next step for this research.^16^ Furthermore, future research examining cognitive differences by gender and occupation conducted within countries would benefit from considering minority status as an additional intersecting stratum, which we were unable to do here. Finally, although an advantage of MAIHDA is that it can accommodate small strata when estimating variance components, the statistical precision of predicted GCF scores for strata with smaller sample sizes is limited (e.g., South African women in Level 3+ occupations, *n* = 10).

Despite the limitations, our study provides valuable insights. Inequities in cognitive function across intersectional strata defined by gender and main lifetime occupational skill across countries were not fully explained by the individual contributions of these factors, suggesting the presence of intersectional effects. This finding may signal important societal processes operating at the country level that differentially influence how gender and occupation shape later‐life cognition. Future studies building on this work will help highlight the roles of structural gender equality, country‐level gender norms, and diverse gendered work experiences in shaping cognitive aging and inform policies aimed at reducing occupational and gender‐based inequalities to improve cognitive health across the life course globally.

## CONFLICT OF INTEREST STATEMENT

The authors declare no conflicts of interest. Any author disclosures are available in the Supporting Information.

## CONSENT STATEMENT

This secondary analysis did not require institutional review board approval because it uses publicly available, anonymized data. For e study used in these analyses, informed consent was obtained from all participants. We followed all ethical standards concerning data privacy and confidentiality, ensuring that individual participants cannot be re‐identified and that the findings are presented in a manner that preserves participant anonymity. This study adheres to the ethical standards of the Declaration of Helsinki.

## Supporting information



Supporting Information

Supporting Information
