# Supplementary material for: Later‐life cognitive function at the intersection of gender and occupation across Harmonized Cognitive Assessment Protocols (HCAPs) in the United States, Chile, Mexico, India, and South Africa
Source: Alzheimers Dement. 2025 Nov 28;21(11):e70923. doi: 10.1002/alz.70923 (PMC12661120; doi:10.1002/alz.70923)
Supplement: Supplementary file 2 — Supporting Information [file ALZ-21-e70923-s002.pdf]

Supplementary Appendix

Measuring and defining lifetime occupational skill level

Main lifetime occupation was self-reported in all studies except Chile where it was determined as the most frequently reported occupation across surveys. According to ISCO-08[26,34]:

- **Level 1** occupations include routine manual and physical labor such as cleaners and helpers, agricultural, forestry and fishery laborers, mine and construction workers, food preparation assistants, and street vendors. These jobs often involve outdoor environments, heavy physical effort, and substantial exposure to occupational hazards such as dust, chemicals, noise, and extreme temperatures. Such conditions are also coupled with limited job autonomy and economic insecurity.
- **Level 2** occupations occupations encompass roles that involve the operation of machinery, clerical work, and customer service. Examples include bus and truck drivers, mobile plant operators, assemblers in manufacturing, accounting and numerical clerks, and shop assistants. Many of these occupations, particularly those in transportation, construction, and manufacturing, are associated with chemical, physical and environmental exposures (e.g., solvents, fumes, repetitive strain). By contrast, clerical and sales roles are less hazardous physically but often characterized by low control, high demands, and psychosocial stressors.
- **Level 3+** occupations require advanced technical knowledge, specialized training, or higher education. Representative roles include science and engineering associate professionals, health professionals and associate professionals (e.g., nurses, laboratory technicians, doctors), teaching professionals, legal and business administrators, computer analysts, and shop or operations managers. These occupations are primarily indoor and office-based, often involving cognitively demanding tasks, decision-making, and creativity. Although they typically provide greater opportunities for cognitive stimulation, they are also associated with risks such as sedentary work, prolonged screen exposure, and psychosocial stressors linked to organizational hierarchies and performance pressures.
- **Never worked** was created as a category for individuals who reported never engaging in paid work, a status not captured in ISCO-08. This category represents an important dimension of lifetime socioeconomic position, particularly among women in many contexts, and reflects long-term differences in both exposure to occupational hazards and access to cognitively enriching work environments

Supplementary Tables and Figures

Table A1: Summary statistics

	USA		Mexico		India		South Africa		Chile	
	Men	Women	Men	Women	Men	Women	Men	Women	Men	Women
Cognitive score, mean (sd)	0.0	0.1	-0.7	-0.9	-1.2	-1.7	-1.1	-1.3	-0.5	-0.5
	(0.9)	(1.0)	(1.0)	(1.0)	(0.8)	(0.8)	(0.8)	(0.8)	(0.9)	(0.9)
Age, mean (sd)	75.7	75.9	68.5	67.5	69.9	69.5	69.4	68.1	70.6	71.0
	(7.1)	(7.2)	(8.9)	(9.0)	(7.3)	(7.9)	(10.5)	(11.6)	(8.1)	(8.4)
Skill level of occupation, %										
Level 1	9.7	3.4	42.8	32.8	20.4	12.9	59.7	46.6	15.8	29.1
Level 2	50.4	57.8	41.1	28.4	64.0	27.6	26.3	6.8	69.2	32.9
Level 3+	37.1	33.4	13.9	9.1	9.6	1.7	7.6	3.0	12.9	10.7
Never worked	2.8	5.4	2.2	29.7	5.9	57.7	6.4	43.5	2.1	27.3
Schooling attainment, %										
None	2.3	3.2	47.1	53.1	50.9	79.0	79.6	81.7	27.3	30.6
Primary	5.2	4.7	22.8	21.5	15.1	9.1	10.3	8.6	29.5	28.8
Lower secondary	6.9	9.8	13.9	17.0	10.0	3.8	5.0	4.7	15.1	15.9
Upper secondary	51.2	55.5	4.6	2.0	17.0	6.5	2.5	2.9	17.9	16.4
Any college	34.3	26.8	11.6	6.5	7.0	1.7	2.6	2.0	10.2	8.2
Parental education, %										
No	7.6	9.1	69.8	70.6	81.4	81.2	89.2	83.4	42.8	45.5
Yes	86.2	85.5	19.4	21.1	16.3	16.0	10.8	16.1	37.9	39.6
Missing	6.2	5.4	10.7	8.3	2.3	2.8	0.0	0.6	19.2	14.9

Data Sources: South Africa - Health and Ageing in Africa: A Longitudinal Study in South Africa; HCAP, Harmonized Cognitive Assessment Protocol; US - Health and Retirement Study; INDIA Longitudinal Aging Study in India - Diagnostic Assessment of Dementia; Mexico - Mexican Health and Aging Study Cognitive Aging Ancillary Study; Chile - Chile Harmonized Cognition Protocol. Estimates include sampling weights.

Figure A1: Flowchart of sample selection

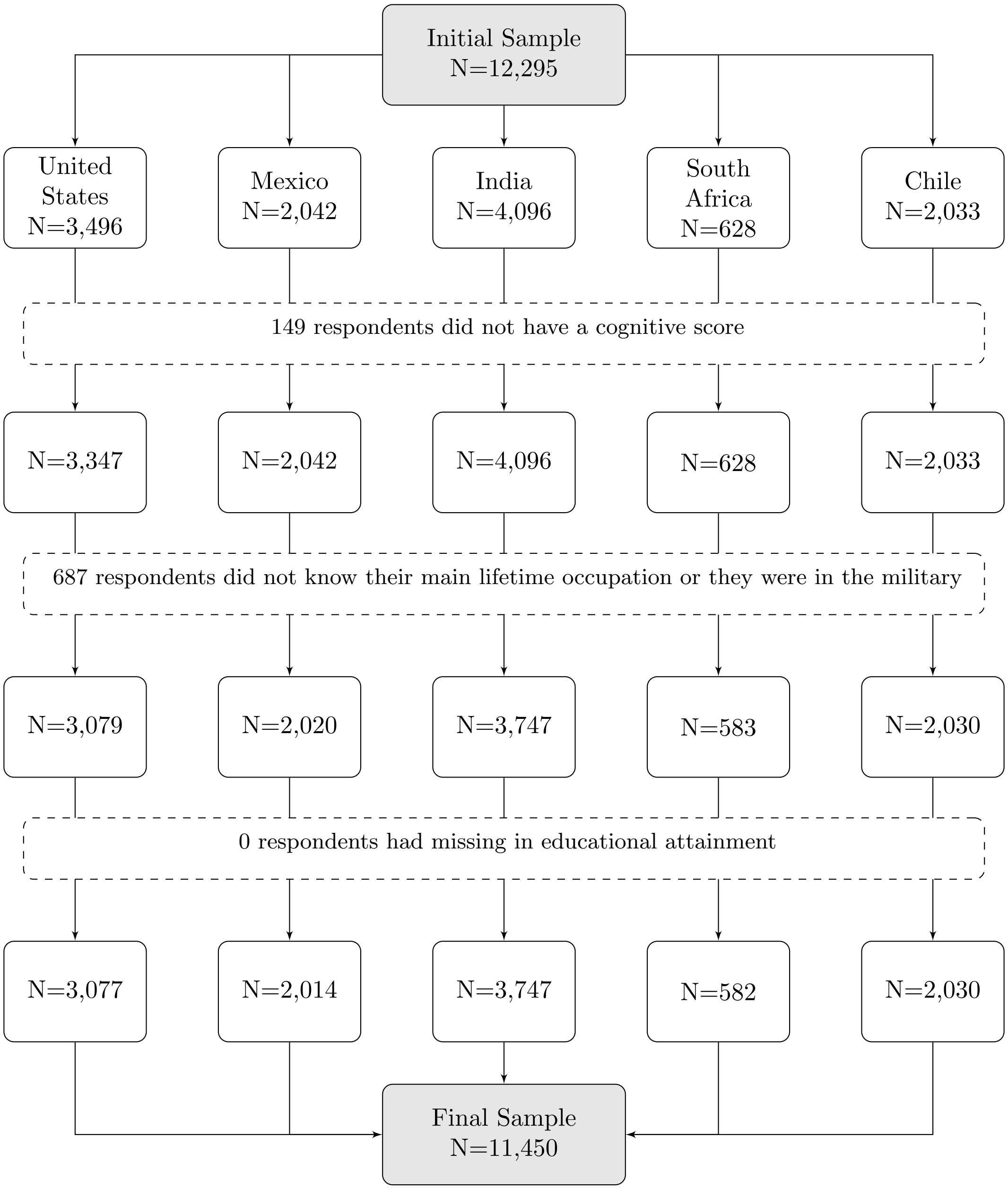

Table A2: Summary of Predicted Cognitive Factor Scores by Stratum

Strata	Predicted Outcomes	Approximate 95% CI
India-W-L1	-1.813	[-1.89, -1.73]
India-W-L2	-1.750	[-1.81, -1.69]
Chile-M-Never	-1.735	[-2.10, -1.43]
India-W-Never	-1.623	[-1.67, -1.57]
South Africa-W-Never	-1.421	[-1.55, -1.30]
South Africa-M-Never	-1.359	[-1.71, -0.97]
India-M-Never	-1.356	[-1.51, -1.18]
South Africa-W-L2	-1.347	[-1.66, -1.03]
South Africa-W-L1	-1.297	[-1.41, -1.17]
India-M-L1	-1.264	[-1.35, -1.18]
Mexico-M-Never	-1.263	[-1.69, -0.83]
Mexico-W-L1	-1.243	[-1.33, -1.15]
India-M-L2	-1.209	[-1.26, -1.16]
South Africa-M-L1	-1.204	[-1.33, -1.09]
Mexico-W-Never	-1.142	[-1.23, -1.04]
Mexico-M-L1	-1.098	[-1.19, -1.00]
South Africa-M-L2	-0.981	[-1.19, -0.78]
Chile-W-Never	-0.887	[-0.99, -0.78]
Chile-M-L1	-0.840	[-0.98, -0.68]
United States-W-Never	-0.718	[-0.90, -0.53]
Chile-W-L1	-0.693	[-0.79, -0.59]
United States-M-Never	-0.630	[-0.90, -0.35]
Mexico-W-L2	-0.628	[-0.74, -0.52]
Mexico-M-L2	-0.614	[-0.71, -0.51]
South Africa-W-L3	-0.570	[-1.13, -0.07]
United States-W-L1	-0.550	[-0.77, -0.31]
Chile-M-L2	-0.545	[-0.62, -0.47]
India-M-L3	-0.535	[-0.64, -0.43]
South Africa-M-L3	-0.452	[-0.80, -0.06]
India-W-L3	-0.324	[-0.60, -0.04]
Chile-W-L2	-0.266	[-0.35, -0.19]
United States-M-L2	-0.215	[-0.29, -0.14]
United States-M-L1	-0.142	[-0.30, 0.01]
United States-W-L2	0.039	[-0.02, 0.10]
Mexico-M-L3	0.051	[-0.10, 0.19]
Chile-M-L3	0.180	[0.03, 0.32]
Mexico-W-L3	0.180	[0.03, 0.32]
Chile-W-L3	0.200	[0.05, 0.35]
United States-M-L3	0.385	[0.31, 0.46]
United States-W-L3	0.448	[0.37, 0.52]

Gender: W = Women, M = Men

Occupation Levels: L1 = Level 1, L2 = Level 2, L3 = Level 3 or 4, NR = Never Worked

Notes: Confidence intervals were estimated using bootstrap methods.

Table A3: MAIHDA model results for Harmonized Cognitive Factor Scores

	Null	Main
Country (ref: USA)		
Mexico		–0.249***
		(0.064)
India		–0.660***
		(0.074)
South Africa		–0.349***
		(0.084)
Chile		–0.196*
		(0.086)
Female		–0.004
		(0.047)
Skill level of lifetime occupation (ref: never worked)		
Level 1		0.134
		(0.076)
Level 2		0.232**
		(0.077)
Level 3+		0.353***
		(0.079)
Schooling attainment (ref: no formal schooling)		
Primary	0.538***	0.538***
	(0.034)	(0.034)
Lower secondary	0.763***	0.763***
	(0.047)	(0.047)
Upper secondary	1.042***	1.041***
	(0.057)	(0.057)
Any college	1.349***	1.349***
	(0.061)	(0.061)
Parental education (ref: no formal schooling)		
Some formal education	0.145***	0.145***
	(0.021)	(0.021)
Missing information	–0.065*	–0.065*
	(0.029)	(0.029)
Age	0.051**	0.051**
	(0.019)	(0.019)
Age^2	–0.001***	–0.001***
	(0.000)	(0.000)
Minority group	–0.265***	–0.265***
	(0.045)	(0.045)
Constant	–1.777**	–1.664**
	(0.605)	(0.608)
Between group variance	0.086	0.022
Within group variance	0.449	0.449
Variance Partition Coefficient (%)	16.018	4.574
Proportional Change of the Variance (%)		75
N	11.370	11.370

Sources: Health and Retirement Study, Mexican Cognitive Aging Ancillary Study, Longitudinal Aging Study in India-Diagnostic Assessment of Dementia, Health and Aging in Africa: Longitudinal Studies in South Africa - HCAP battery and Chile Cog. Harmonized score of global cognitive function was standardized to the distribution of the unweighted HRS-HCAP sample, which had a mean of 0 and a standard deviation of 1. The sample size is lower than the analysis sample due to individuals with missing minority status. The minority group variable was classified in the US according to the race/ethnicity groups of non-Hispanic Black, Hispanic, and Other ("minority") and non-Hispanic White ("nonminority"); in Mexico as rural ("minority") and urban region of residence ("nonminority"); in India according to caste as Scheduled Caste or Scheduled Tribe ("minority") and Other Backward Class or Other or no caste group ("nonminority"); in Chile as belonging to any indigenous group ("minority") or being non-indigenous ("nonminority") and in South Africa according to country of birth as Mozambique or other ("minority") and South Africa ("nonminority")
+ $p < 0.1$ * $p < 0.05$, ** $p < 0.01$, *** $p < 0.1$
